# Uptake and availability of new outpatient cancer medicines in 2010–2021 in Nordic countries – survey of competent authorities

**DOI:** 10.1186/s12913-023-10421-x

**Published:** 2023-12-18

**Authors:** Kati Sarnola, Hanna Koskinen, Katariina Klintrup, Cecilie Astrup, Terhi Kurko

**Affiliations:** 1 Research Unit, Social Insurance Institution of Finland (Kela), P.O. Box 450, Helsinki, 00056 KELA Finland; 2 Medical Advisory Centre, Social Insurance Institution of Finland (Kela), Helsinki, Finland; 3 Business Intelligence and Health Economy, Amgros I/S, Copenhagen, Denmark

**Keywords:** Cancer medicines, Distribution, Costs, Uptake, Managed entry agreements, Availability, Nordic countries, Survey, Orally administered cancer medicines

## Abstract

**Background:**

Nordic countries excel in cancer care, but studies on uptake, costs, or managed entry agreements of cancer medicines have not been conducted recently. The aim of this study was to examine the uptake and availability of orally administered new cancer medicines in Nordic countries. Orally administered cancer medicines enable and are used in the community as part of outpatient care. Firstly, we studied the distribution, costs and adoption of managed entry agreements of these medicines, and secondly, uptake of and managed entry agreements for cancer medicines used in outpatient care that were granted marketing authorization in Europe in 2010–2021.

**Methods:**

An E-mail survey of competent authorities, meaning pharmaceutical service organizers, payers or other government or non-government actors developing pharmaceutical service operations, in Denmark, Finland, Iceland, Norway, and Sweden in April-June 2022. The data were analysed using frequencies and percentages for descriptive analysis.

**Results:**

The distribution of cancer medicines has similarities in Finland, Iceland, Norway, and Sweden, where cancer medicines can be distributed both via hospitals or hospital pharmacies for inpatient use, and via community pharmacies for outpatient use. In Denmark, cancer medicines are predominantly distributed via publicly funded hospitals. In all countries that provided data on the costs, the costs of cancer medicines had notably gone up from 2010 to 2021. The number of reimbursable medicines out of new cancer medicines varied from 36 products in Denmark and Iceland to 51 products in Sweden, out of 67 studied products. Managed entry agreements, often with confidential discounts, were in use in all Nordic countries. The number of agreements and the cancer types for which agreements were most often made varied from three agreements made in Iceland to 35 agreements made in Finland, out of 67 studied products. Average days from authorization to reimbursement of new cancer medicines varied from an average of 416 to 895 days.

**Conclusions:**

Nordic countries share similar characteristics but also differ in terms of the details in distribution, adopted managed entry agreements, market entry, and availability of new orally administered cancer medicines used in the outpatient care. The costs of cancer medicines have increased in all Nordic countries during the last decade. Due to differences in health care and because orally administered cancer medicines can be dispensed at community and hospital pharmacies in all studied countries other than Denmark, the number of reimbursable medicines and managed entry agreements vary between countries. However, Nordic countries show good agreement for 2010 to 2021 in entry and reimbursement decisions of novel cancer medicines.

**Supplementary Information:**

The online version contains supplementary material available at 10.1186/s12913-023-10421-x.

## Introduction

### Evolved cancer care has led to rapidly growing costs

Cancer medicines are one of the fastest growing and evolving therapeutic areas in the pharmaceutical industry [1]. Novel cancer medicines form approximately a third of all new compounds in the medicine pipeline and the largest number of new marketing authorizations in comparison to other therapeutic areas [1, 2].

Development and authorization of novel cancer medicines has led to significant improvements in cancer therapy [3]. Molecular characterization of tumours has enabled cancer therapy to shift from nonspecific cytotoxic treatments to targeted therapies with small-molecule inhibitors and monoclonal antibodies. This so-called personalised cancer therapy, or precision oncology, may improve outcomes and quality of life in multiple cancer types [4]. Therapies have become available for smaller subpopulations and different stages of the disease, and enable solutions for medicine resistance [5, 6]. Furthermore, many novel therapies can be used in outpatient care based on their oral administration route. This facilitates cancer care outside of hospitals as with chronic diseases that can be treated from home.

### Pricing, uptake, and patient access to cancer medicines

Patient access to medicines can be measured in multiple ways [7]. According to the OECD report in 2020 [7], *availability* (covering marketing authorization, country launches and elapsed time between the authorization and the launch), *affordability* (that considers the coverage and co-payments of medicines), and *accessibility* (covering the ability of patients to obtain the medicines but also the factors related to availability and affordability) should all be taken into account. All in all, according to the European Cancer Organization and the opinions from the public hearing of the European Parliament, people have a right to equal access to affordable and optimal cancer care [8, 9].

In Europe, cancer medicines are authorized with centralised procedures, meaning the marketing authorization is granted simultaneously for all European Union Member States as well as the European Economic Area (EEA) countries Iceland, Liechtenstein and Norway [10]. However, decisions on pricing, reimbursement and uptake are done at national level, and patient access to new medicines is not only dependent on the licensing decision but also on the national uptake decision [11]. In practice, patient access to medicines is limited if high-cost medicines are not covered by a publicly funded health care system [12, 13]. Several studies have reported country-to-country variation in market entry [11–14], time to entry [13] and costs [15] of novel cancer therapies, although it should be noted that studies vary in their focus, methods, restrictions and their criteria for inclusion. Overall, price regulation, smaller market size, and low expected prices were reported to correlate with longer time between the first global launch and individual country launches [13], although differences have also been reported between countries with similar purchasing power [16]. New Clinical Trial regulation aiming to harmonize the submission, assessment, and supervision processes for clinical trials in the European Union has been applied since January 2022 [17]. Furthermore, the regulation on Health Technology Assessment contributes to improving availability for EU patients of innovative technologies and ensuring an efficient use of resources across the Union [18]. The regulation applies as of January 2025. It is to be seen how these will translate to national pricing, reimbursement, and uptake decision-making. As of today, product-level evaluations on the benefits and added clinical value are produced and made publicly available by the French National Authority for Health [19], for example.

In addition to optimizing national uptake procedures, the European Medicines Agency and European countries try to manage with the unmet medical needs of cancer patients by authorizing medicines in earlier phases of their development. Thus, recent studies have progressively focused on the evidence on launched cancer medicines [20–25]. Studies suggest that the evidence for launched therapies is often scarce, and especially lacking on overall survival and health-related quality-of-life benefits, due to non-controlled settings and the use of surrogate endpoints. To manage the uncertainty, but also to promptly provide treatments to patients in need, managed entry agreements between pharmaceutical companies and health care payers are common [26]. Differences in the assessment of evidence and diverse decision-making on access and reimbursement may pose a risk for different patient access of vital therapies in different countries [11, 27]. The new Clinical Trials Regulations aim to harmonize this decision-making [17]. In regard to cancer medicines, significant uncertainty is associated with early access decisions due to limited clinical and cost-effectiveness data. Managed entry agreements are seen as common policy tools in many European countries [28], but concerns have been raised because of the lack of transparency of such agreements [29]. Systematic research on managed entry agreements of cancer medicines is, to our best knowledge, lacking.

### Nordic countries excel in cancer care, but face economic challenges

The Nordic countries, Iceland, Denmark (excluding Greenland), Finland, Norway, and Sweden have common geographical location and common heritage in organizing health care. Nordic countries have a similar population base and a tax-based, locally administrated, high-in-quality health care system in which all citizens have equal access to services, and national health coverage [30, 31]. Nordic countries excel in cancer care, being among the countries with the highest cancer survival both in Europe and globally [32, 33]. Despite the growing incidence of cancer, mainly due to demographic changes and improved cancer detection, mortality rates have remained stable or even decreased. With the implementation and regular updates of national and European guidelines including, for example, the European Society for Medical Oncology (ESMO) guidelines, cancer patients in the Nordic countries receive high-quality care according to the best clinical practices. Cancer prevalence and survival are similar in all Nordic countries [33, 34].

In the Nordic countries, similarly to many other countries, both direct costs of cancer and costs of cancer medicines have increased over time [32, 35]. At the same time, there has been the shift in care from inpatient setting to outpatient care [35]. According to a comparison published in 2022, in Denmark and Finland inpatient care costs have decreased, while in Norway and Sweden inpatient care costs have increased, although the number of inpatient days has decreased everywhere, indicating differences in health policy and arranging health care [36]. To understand the reasons behind the changes in the cost structure, timely and detailed research on the topics of costs and uptake of cancer medicines is needed.

### Knowledge gap and the aims of this study

Studies on uptake, costs or managed entry agreements of cancer medicines have not recently been conducted in Nordic countries, although earlier research supports country-to-country variation in market entry. The aim of this study was to examine the uptake and availability of orally administered new cancer medicines. Firstly we studied the distribution, costs, and adoption of managed entry agreements of these medicines, and secondly, the uptake of and adopted managed entry agreements for cancer medicines used in outpatient care, for medicines that were granted marketing authorization in Europe in 2010–2021.

## Materials and methods

The study was executed as an e-mail survey of competent authorities in Nordic countries and consisted of two parts. The first part consisted of fifteen general questions on the distribution, costs, and uptake of new cancer medicines in the respondent’s country. The latter part consisted of product-level questions on a total of 67 products on the uptake (including the reimbursement status) and possible managed entry agreements of cancer medicines that were granted marketing authorization in Europe in 2010–2021 (Supplementary Material 2) and were suitable for outpatient care based on the self-administrable oral route. In this context, managed entry agreements included all types of risk-sharing and managed entry agreements. For this survey, cancer medicines were defined as those affecting the tumor and classified to L01, L02 and individual ATC-codes L04AX02, L04AX04, and L04AX06 in Anatomical Therapeutic Chemical (ATC) classification [37]. The survey form was developed with the expertise of the research group (KS, TK, KK and HK) and on the earlier publication on the market entry and reimbursement status of cancer medicines in Finland [38].

The survey form was piloted in Denmark in April 2022 to ensure its usability. Minor modifications, such as adding the sections for description of the medicines distribution system and for additional comments, were made, and the data received from piloting were included in the final study material. Respondents representing competitive authorities from Iceland, Norway, and Sweden were selected with convenience sampling based on the recommendations from the Danish respondent (CAF). They represented pharmaceutical service organizers, payers or other government or non-government actors developing pharmaceutical service operations, in order to provide a broad picture on the studied topic in the selected countries. The survey for the respondents in remaining countries was executed in April-June 2022. Consequently, respondents represented The National University Hospital of Iceland (Landspítali) in Iceland, Amgros in Denmark, The Social Insurance Institution of Finland (Kela) in Finland, Sykehusinnkjøp HF in Norway, and The Swedish Association of Local Authorities and Regions (SALAR) in Sweden. Respondents were instructed to consult colleagues, if necessary, when they filled the survey. Respondents were given the time they needed to participate, and they were reminded when necessary. Finnish authors (KS, TK and HK) were responsible for providing answers from Finland. Before submitting the manuscript, respondents were given the opportunity to read and comment on the manuscript.

The data were analysed using Microsoft Excel, and frequencies and percentages for descriptive analysis were produced. Uptake and possible managed entry agreements made for these medicines were also studied according to cancer types. Cancers were classified as haematological, lung, breast, prostatic, and other cancer medicines, the latter including medicines with multiple indications and/or indications other than haematological malignancies, or lung, breast, or prostate cancer, for example GI tract, bowel or liver cancer.

## Results

All five Nordic countries provided answers during the survey period of 9 weeks in April-June 2022 and provided supplementary information in August 2022, at the latest. The results of the survey are presented (in the following chapters) as reported by the respondents.

### Distribution, costs, and adoption of managed entry agreements of cancer medicines

Distribution of cancer medicines varies between Nordic countries, although similarities can be found. In Finland, Iceland, Norway, and Sweden, cancer medicines are distributed both via hospitals or hospital pharmacies and via community pharmacies.

In practice, in Iceland, for example, if a cancer medicine needs to be administered at a clinic, the medicines are administered at clinics affiliated with government hospitals, while orally or subcutaneously administrable medicines are distributed via hospital or community pharmacies. The practice is very similar to Finland, where medicines for inpatient care that are administered in public regional hospitals are provided by hospital pharmacies affiliated to the hospitals, and medicines for outpatient care are mainly covered from national health insurance funds, distributed via private-owned community pharmacies, and administered at home by the patient.

In Norway, on the other hand, there are three levels in the health care system: the Central State, four Regional Health Authorities and the municipalities. The State provides policies and legislation and allocates the funds, while the Regional Health Authorities provide the health care services. These services can be provided either by hospitals (treatment in the hospital), or by community pharmacies (medicines dispensed by community pharmacies, so called “H-prescriptions”).

Distribution of cancer medicines in Denmark differs from other Nordic countries. In Denmark, cancer medicines are predominantly distributed via publicly funded hospitals. The procurement of medicines is managed by Amgros. Reimbursement via national health funds or by other means are not in use; instead, the Danish Medicines Council approves a product as a standard treatment. The Danish Medicines Council was established in 2017 and prior to that Denmark did not have a prioritization system, which means that most medicines were reimbursed before 2017 as they automatically were available at public Danish hospitals if the pharmaceutical company chose to launch in Denmark.

In all countries reporting cost data, the total costs of all prescription medicines and of cancer medicines had notably gone up from 2010 to 2021 (Table 1). In the comparison from 2010 to 2021 in each country, the increase in the total costs was highest in Denmark (135%) and lowest in Finland (35%), while the increase in cancer medicines’ costs was highest in Norway (239%) and lowest in Finland (135%).

**Table 1:** Costs of all prescription and cancer medicines (ATC-groups L01, L02 and individual ATC-codes L04AX02, L04AX04, L04AX06) in Nordic countries. Inflation omitted. Data from Iceland was not available

	Denmark	Finland	Norway^a^	Sweden
Costs of all prescription medicines in 2010	935 million euros	1 701 million euros	977 million euros	n/a
Costs of all prescription medicines in 2021	2.2 billion euros	2.3 billion euros	1.6 billion euros	3.4 billion euros
Costs of all prescription medicines per capita in 2021^b^	371.7 euros per capita	414.7 euros per capita	291.7 euros per capita	319.9 euros per capita
Increase in costs of all prescription medicines from 2010 to 2021	135%	35%	64%	n/a
Costs of cancer medicines in 2010	226 million euros	345 million^c^ euros	89 million euros	n/a
Costs of cancer medicines in 2021	708 million euros	813 million^c^ euros	302 million euros	421 million euros
Share of the costs of cancer medicines in 2021 of the costs of all prescription medicines in 2021^b^	32%	35%	19%	12%
Costs of cancer medicines per capita in 2021^d^	120.4 euros per capita	146.7 euros per capita	55.6 euros per capita	39.9 euros per capita
Increase in costs of cancer medicines from 2010 to 2021	213%	135%	239%	n/a
Costs of cancer medicines used in the outpatient care in 2010	n/a	93 million euros	43 million euros^e^	n/a
Costs of cancer medicines used in the outpatient care in 2021	n/a	297 million euros	143 million euros^e^	396 million euros^f^
Increase in costs of cancer medicines used in outpatient care from 2010 to 2021	n/a	219%	233%	n/a

^a^Costs are given in AIP, excluding VAT and pharmacy markup; data from 2020 (year 2021 not available)

^b^Share is calculated based on the costs of cancer medicines and the costs of all prescription medicines 2021, regardless of the fact that depending on the country, numbers can be presented with or without VAT or pharmacy markup, and some cancer products may be dispensed via hospitals or via other inpatient route, meaning those costs might not be allocated as costs of all prescription medicines

^c^Sales in wholesale prices excluding VAT and pharmacy markup

^d^Population data based on the data of World Population Review (2022) in 2022

^e^Only H-prescriptions (only including costs for medicines the patient administers at home)

^f^Not including Dalarna and Blekinge

Managed entry agreements are in use in all Nordic countries. Agreements are made solely for high-cost inpatient care medicines in Iceland and mainly for inpatient care medicines in Denmark. The agreements for outpatient care medicines were adopted in Sweden in 2014, followed by Denmark and Finland in 2017. In Norway, agreements have been made for a long time (although the exact year was not reported) and revisions to the system have been conducted in 2013 and 2020. Similarly, the exact year of the adoption of agreements in Iceland was not available. In some countries, agreements are made for both inpatient and outpatient care medicines, while in other countries agreements are only done for hospital medicines.

### Uptake of and managed entry agreements made for authorized cancer medicines used in the outpatient care

**Fig. 1:**
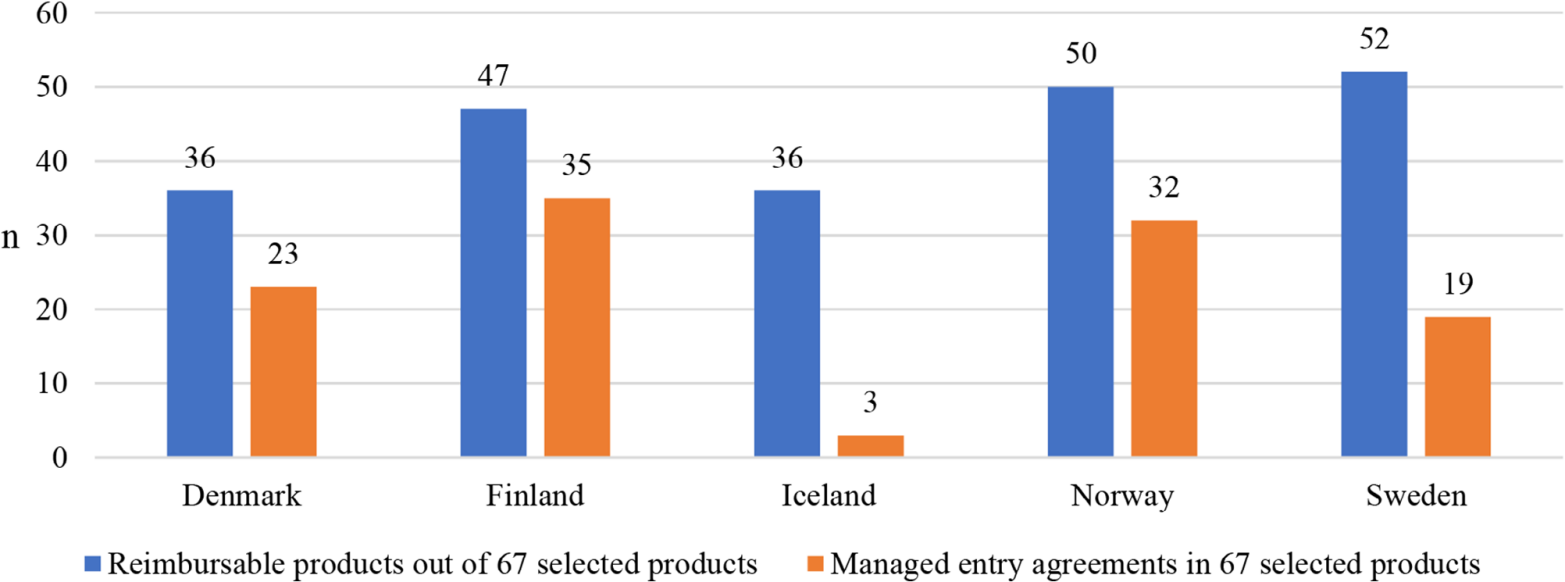
Number of reimbursable medicines out of 67 cancer medicines and adopted managed entry agreements for those medicines in Nordic countries in 2010–2021

A total of 67 cancer medicines suitable for outpatient care had been granted a marketing authorization in Europe from 2010 to 2021. The number of reimbursable medicines and managed entry agreements made varied from country to country (Supplementary Table 1). Out of the 67 medicines, the number of reimbursable medicines was highest in Sweden (n = 51) and lowest in Denmark and Iceland (n = 36) (Fig. 1). A total of 46 medicines were reimbursed in at least three Nordic countries and 26 were reimbursed in all Nordic countries. There were a total of 10 medicines that were not reimbursed in any of the countries at the time of this analysis. Nine of these medicines were only authorized in 2020 or 2021, indicating that their reimbursement processes may not have been finalised yet.

The number of managed entry agreements made was highest in Finland (n = 35) and lowest in Iceland (n = 3), indicating Iceland lags behind the other Nordic countries in terms of the number of agreements. In Nordic countries, managed entry agreements for outpatient care medicines were mainly agreements where a confidential discount was granted by the marketing authorization holder.

**Fig. 2:**
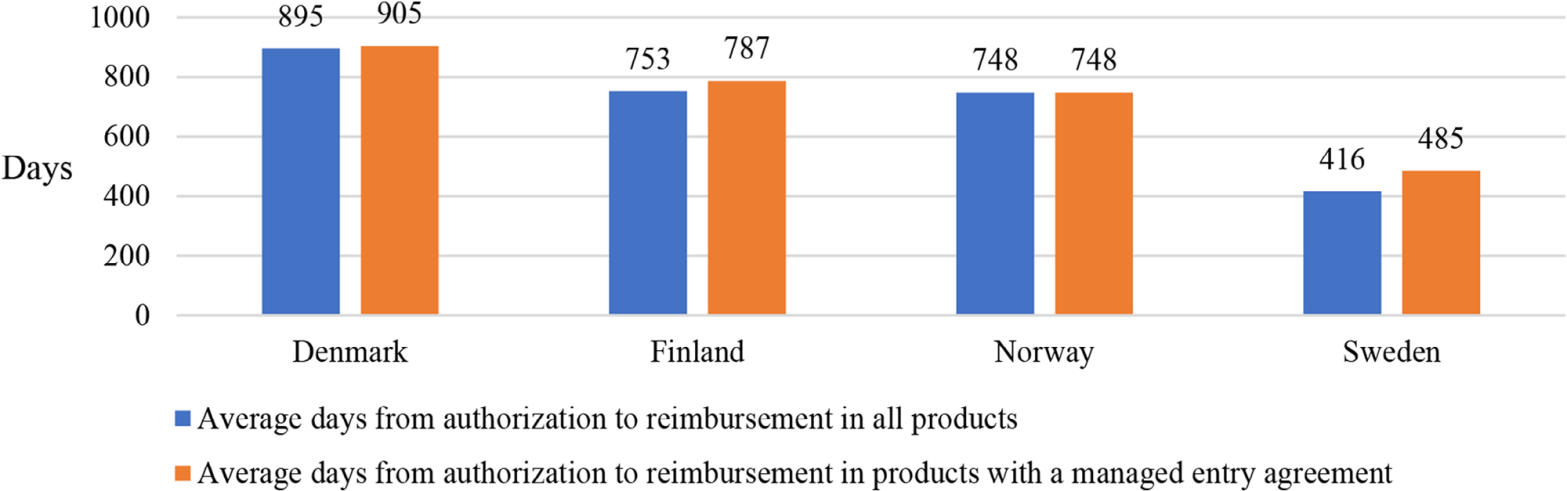
Average days from marketing authorization to reimbursement in all 67 selected medicines and of those with a managed entry agreement in Denmark, Finland, Norway, and Sweden in 2010–2021. Information on the reimbursement dates was not provided from Iceland, and was only partially reported from Denmark, Norway, and Sweden

From 2010 to 2021, days from authorization to reimbursement of those medicines varied from an average of 416 days in Sweden to 895 days in Denmark (Fig. 2). Delay from authorization to reimbursement with a managed entry agreement was slightly longer than the delay from authorization to reimbursement of all medicines in all countries except Norway, where the delay was the same. Information on the reimbursement dates was not provided from Iceland.

**Fig. 3:**
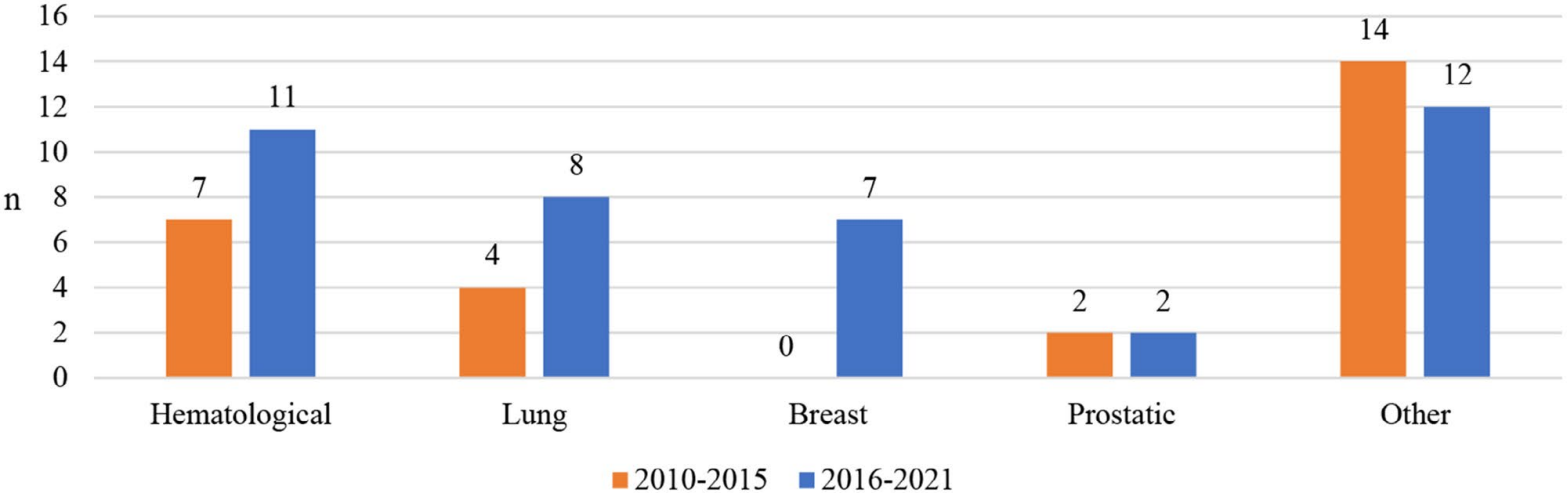
The classification of 67 authorized outpatient care cancer medicines based on the cancer type in 2010–2015 and in 2016–2021

Uptake and possible managed entry agreements made for these medicines were also studied according to cancer types. Out of the 67 studied medicines, the majority were haematological and lung cancer medicines and medicines classified for other cancers (Fig. 3). However, the number of medicines aiming to treat other cancers was slightly decreasing, while the number of medicines for haematological, lung and especially breast cancer had increased in 2016–2021 in comparison to 2010–2015.

**Fig. 4:**
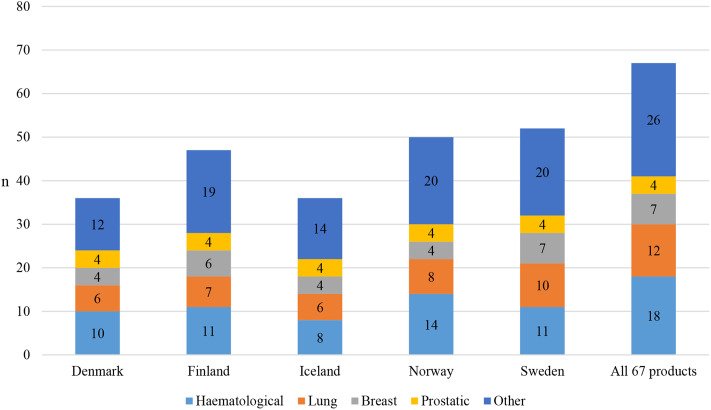
The number of reimbursable outpatient care cancer medicines according to the cancer type out of the 67 medicines in Nordic countries in 2010–2021

The number of reimbursable medicines according to the cancer type out of the number of reimbursable medicines in each country was somewhat even among Nordic countries (Fig. 4). For example, all four prostatic cancer medicines were reimbursed in Nordic countries, except for abiraterone in Denmark, where medicines can be distributed via public hospitals (Supplementary Table 1).

Some differences in the reimbursement between individual medicines could be detected (Fig. 4). For example, from the two tumour-agnostic therapies, larotrectinib is reimbursed only in Finland and Sweden, whereas entrectinib is reimbursed in all Nordic countries. Similarly for second-generation EGFR-TKI medicines, afatinib is reimbursed in Finland, Norway, and Sweden, whereas osimertinib is reimbursed in all Nordic countries.

**Fig. 5:**
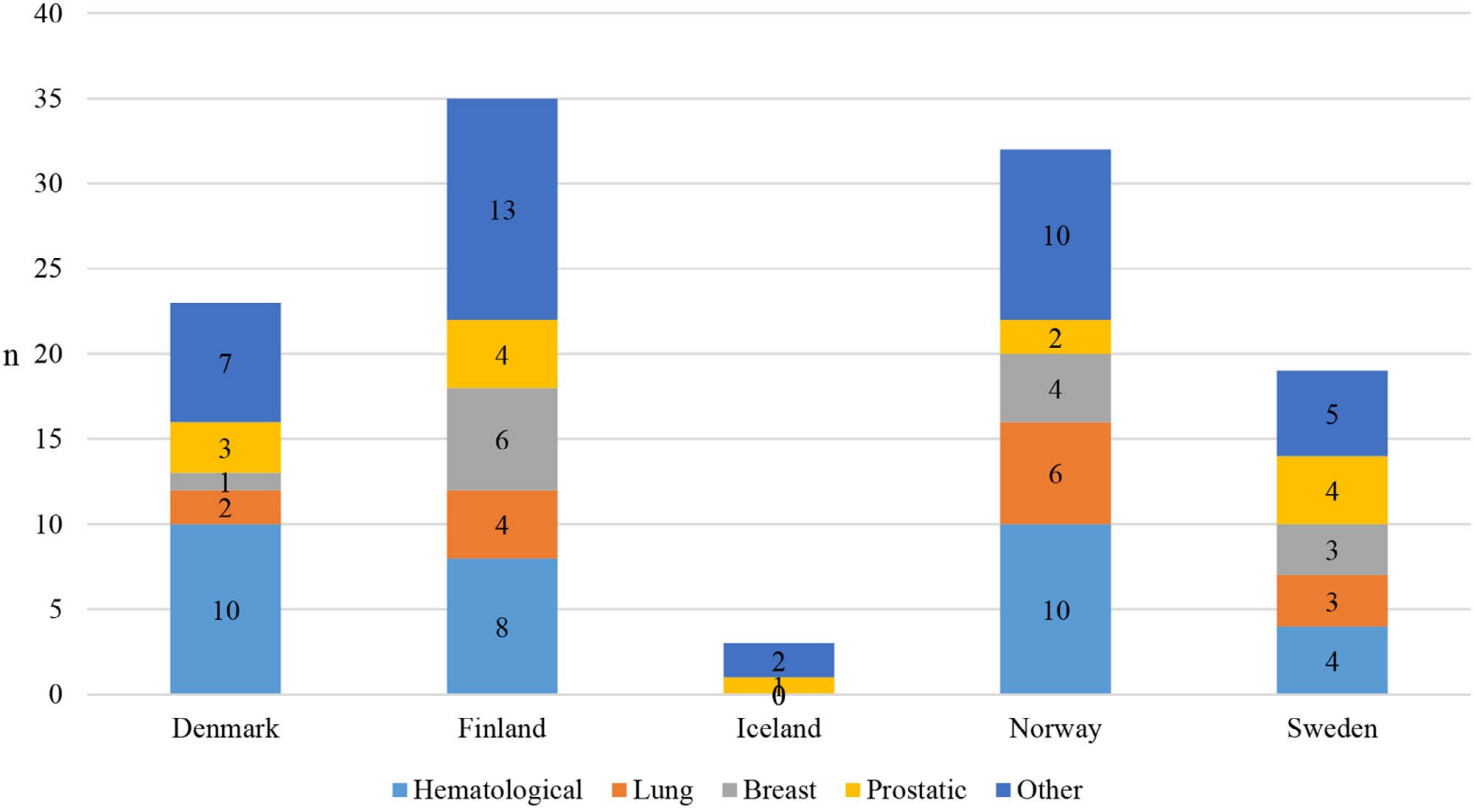
The number of adopted managed entry agreements out of the 67 outpatient care cancer medicines according to the cancer type in Nordic countries. In Iceland, managed entry agreements were made for prostatic and other cancer medicines

The numbers of managed entry agreements of medicines in each cancer type varied among Nordic countries (Fig. 5). For the four prostatic cancer medicines, managed entry agreements were made for all reimbursed medicines in Finland, Sweden, and Denmark. For lung cancer medicines, agreements were most commonly made in Norway (6 agreements) in comparison to the other countries (0–4 agreements). Similarly, agreements were most commonly made for breast cancer medicines in Finland (6 agreements) in comparison to the other countries (0–4 agreements). For example, managed entry agreements were common with CDK4/6 inhibitors in all Nordic countries but agreements for neratinib and alpelisib were only made in Finland.

## Discussion

This study provides a timely view on the uptake and availability of outpatient care cancer medicines in Nordic countries in the last decade. Nordic countries share similar characteristics but also differ in terms of the distribution, adopted managed entry agreements, market entry and availability of new cancer medicines used in outpatient care.

According to the results from this study, the costs of cancer medicines have increased in Nordic countries during the last decade. The results are consistent with the global data indicating an increase in the use of and spending on medicines globally, and the size of the global medicine market [39]. It should be noted that the cost data of this study are not comparable between countries. Instead, the data present the situation as well as the occurred change in each country in 2010 and 2021. In Finland, for example, the share of medicines that are used in outpatient care and reimbursed via the national reimbursement scheme is high, resulting to high costs in both total and outpatient care costs of cancer medicines (Table 1). On the other hand, the increase in the costs of inpatient care medicines has been reported in both Norway and Sweden [36], reflecting the differences in organizing health care and in the cost structures between inpatient and outpatient care medicines. These differences were also seen in this study: the number of reimbursable medicines per country varied.

Although there are some differences in the number of reimbursable medicines among countries, the Nordics generally agree in their reimbursement and uptake decisions. Most of the 67 studied medicines were reimbursed in three or more countries and a fifth in all countries (Supplementary Table 1). Presumably, Nordic countries excel in the uptake of novel cancer therapies [32, 33, 36]. Although the major purpose of our study was not to evaluate the effectiveness or added benefit of novel therapies, it is obvious that uptake decisions are rarely made solely based on the effectiveness of novel therapies in comparison to existing ones. Although health technology assessment (HTA) and evaluation mechanisms are in use in Nordic countries, of the 26 medicines that were launched in all Nordic countries, eight provide moderate clinical added value when compared with available therapies according to the publicly available data from the French National Authority for Health [40] (Supplementary Table 1). Furthermore, nine medicines out of 26 were evaluated to provide a minor benefit, and eight medicines were evaluated to provide no added benefit when compared with available therapies. One medicine was not recommended for reimbursement at all [19]. As the costs of cancer care are increasing, the importance of effectiveness of novel therapies is likely to increase also.

According to the results, medicines for different cancer types are quite evenly reimbursed in the Nordic countries. In the big picture, a slight shift can be seen. The number of medicines aiming to treat other cancers appears to be decreasing, while the number of medicines for haematological, lung and especially breast cancer has increased in 2016–2021 in comparison to 2010–2015 (Fig. 3), reflecting a shift towards more and more precise, tissue-specific care. This shift has been reported in the previous literature also [4–6]. Of course this shift may partly be induced by differences in patient characteristics and in the disease prevalence between countries. When moving towards more specific care and treatments requiring gene testing, for example, there might be no need for certain medicines in some countries, while the disease and patient population might be more common in others.

The results of this study also show that managed entry agreements, often with confidential discounts, are in use in all Nordic countries. The results are consistent with earlier studies reporting that financial-based agreements are more common in comparison to performance-based agreements due to their simplicity [28], although performance-based agreements appear to become increasingly more common [29]. The number of agreements made in each country varied. This may be due to the differences in the uptake procedures of novel therapies. n Denmark, for example, cancer medicines are predominantly distributed via publicly funded hospitals and managed entry agreements are made mainly for inpatient care medicines. In Finland, on the other hand, medicines can be distributed via inpatient and outpatient routes, but the agreements are made for outpatient care medicines. Furthermore, managed entry agreements for outpatient care medicines have been adopted differently, for example in Sweden from 2014, which may explain differences in the speed of the process: The average days from authorization to reimbursement of novel cancer medicines was lowest in Sweden, followed by Finland and Norway with approximately 300 additional days and Denmark with 500 additional days in comparison to Sweden. Again, differences in organizing health care are likely to affect this. Logically, a managed entry agreement with negotiations seems to lengthen the process in all countries except Norway. The lengths of managed entry agreements were not studied but the following may explain some differences between countries. If a country categorically concluded shorter agreements and then renewed them, it would lead to a higher number of agreements in the long run. In general, price regulation, smaller market size and low expected prices indicate a longer time between the first global launch and individual country launches [13]. As the negotiated prices of the medicines with a managed entry agreement are not publicly available, we were unable to verify the hypothesis of Ferrario et al. [13] on a longer launch time resulting in lower price. In Denmark, however, this hypothesis may not apply. In Denmark, a 14-day negotiation time is applied as the medicines go through the Danish Medicines Council process, and the negotiation does not lengthen the process.

The strength of this study is the new information provided, given that the country-specific information is a valuable source for international comparisons now and in the future [41]. However, this study also has limitations. A major limitation is that providing comparable, uniform information on the research topic, especially with a survey method, turned out to be challenging. Due to the differences in the systems, statistics and in organizing health care in general, we were unable to provide fully comparable information. Yet, the data provides a timely picture on the distribution, costs, and managed entry agreements of novel cancer therapies in each Nordic country. Another limitation is that this study mainly focuses on the medicines used in outpatient care and, thus, does not provide information on inpatient care medicines and on the interplay of inpatient and outpatient care medicines in health care systems. Depending on the country, systems and health care are organized differently and thus, inpatient and outpatient care medicines have a different emphasis for cancer care. Another limitation is that because the medicines were identified manually, it is possible that some medicines may be missing from our data. Regardless of these limitations, this study provides a timely view on the increasing costs and the differences in market entry and availability of cancer medicines in Nordic countries, and the value of this study does not depend on the absence of individual medicines. Further research on the topic is obviously still needed, especially on the comprehensive and in-depth perspectives, considering both inpatient and outpatient care medicines. In addition, more research on the previous and future changes in pharmaceutical policy around the uptake of new medicines, such as the Reform of the EU pharmaceutical legislation, is needed.

## Conclusions

Nordic countries share similar characteristics but also differ in terms of the distribution, adopted managed entry agreements, uptake, and availability of new cancer medicines used in the outpatient care. The costs of cancer medicines have increased in all Nordic countries during the last decade. In the Nordics, cancer medicines can be distributed via inpatient and outpatient routes, and the health care is organised differently. In Finland, Iceland, Norway, and Sweden, cancer medicines are distributed via hospitals or hospital pharmacies for inpatient use, and via community pharmacies for outpatient use, while in Denmark, cancer medicines are predominantly distributed via publicly funded hospitals. This affects the number of reimbursable medicines and the number of adopted managed entry agreements. Furthermore, the average number of days from authorization to reimbursement varied. However, Nordic countries mainly seem to agree in entry and reimbursement decisions of novel cancer medicines.

### Electronic supplementary material

Below is the link to the electronic supplementary material.

**Supplementary Material 1**: Table 1. Reimbursement status, days to reimbursement from marketing authorization and if a managed entry agreement has been made of cancer medicines authorized in 2010–2021 (N = 67) in Nordic countries according to the situation in June–August 2022 (depending on the timing of the response)

**Supplementary Material 2**

## Data Availability

The datasets used and/or analysed during the current study are available from the corresponding author on reasonable request.
