# Supplementary material for: Uptake and availability of new outpatient cancer medicines in 2010–2021 in Nordic countries – survey of competent authorities
Source: BMC Health Serv Res. 2023 Dec 18;23:1437. doi: 10.1186/s12913-023-10421-x (PMC10729379; doi:10.1186/s12913-023-10421-x)
Supplement: Supplementary file 1 — Supplementary Material 1: Table 1. Reimbursement status, days to reimbursement from marketing authorization and if a managed entry agreement has been made of cancer medicines authorized in 2010–2021 (N = 67) in Nordic countries according to the situation in June–August 2022 (depending on the timing of the response) [file 12913_2023_10421_MOESM1_ESM.docx]

Supplementary Table 1. Reimbursement status, days to reimbursement from marketing authorization and if a managed entry agreement has been made of cancer medicines authorized in 2010–2021 (N=67) in Nordic countries according to the situation in June-August 2022 (depending on the timing of the response).

|  | Cancer type | **Denmark** | | **Finland** | | **Iceland** | | **Norway** | | **Sweden** | | **Number of Nordic countries where reimbursed** | **Hauté Autorité de Santé (HAS) evaluation for those reimbursed in all Nordic countries** |
| --- | --- | --- | --- | --- | --- | --- | --- | --- | --- | --- | --- | --- | --- |
| Product name, *active ingredient* in the order of authorization |  | DtR | MEA | DtR | MEA | DtR | MEA | DtR | MEA | DtR | MEA |  |  |
| Authorized in 2021 | | | | | | | | | | | |  |  |
| - Nexpovio, *selinexor* | hematological | NR | No | NR | No | NR | No | NR | No | NR | No | 0 |  |
| - Brukinsa, *zanubrutinib* | hematological | 126 | Yes | NR | No | NR | No | 216 | Yes | NR | No | 2 |  |
| - Copitkra, *duvelisib* | hematological | NR | No | NR | No | NR | No | NR | No | NR | No | 0 |  |
| - Gavreto, *pralsetinib* | lung | NR | No | NR | No | NR | No | NR | No | 217 | No | 1 |  |
| - Koselugo, *selumetinib* | other | NR | No | NR | No | NR | No | NR | No | NR | No | 0 |  |
| - Lumykras, *sotorasib* | lung | NR | No | NR | No | NR | No | NR | No | NR | No | 0 |  |
| - Onureg, *azacitinide* | hematological | NR | No | NR | No | NR | No | NR | No | NR | No | 0 |  |
| - Qinlock, *ripretinib* | other | NR | No | NR | No | NR | No | NR | No | NR | No | 0 |  |
| - Tepmetko, *tepotinib* | lung | NR | No | NR | No | NR | No | NR | No | NR | No | 0 |  |
| Reimbursable products |  | 1 | | 0 | | 0 | | 1 | | 1 | |  |  |
| Managed entry agreements |  | 1 | | 0 | | 0 | | 1 | | 0 | |  |  |
| Average days from authorization to reimbursement |  | 126 | | - | | - | | 216 | | 217 | |  |  |
| Average days from authorization to reimbursement in products with a managed entry agreement |  | 126 | | - | | - | | 216 | | - | |  |  |
| Authorized in 2020 | | | | | | | | | | | |  |  |
| - Ayvakyt, *avapritinib* | other | NR | No | NR | No | NR | No | NR | No | NR | No | 0 |  |
| - Calquence, *acalabrutinib* | hematological | 581 | Yes | 616 | Yes | NR | No | 691 | Yes | 245 | No | 4 |  |
| - Daurismo, *glasdegib* | hematological | NR | No | NR | No | NR | No | NR | No | NR | No | 0 |  |
| - Inrebic, *fedratinib* | hematological | 413 | Yes | NR | No | NR | No | 496 | Yes | NR | No | 2 |  |
| - Nubeqa, *darolutamide* | prostatic | 425 | Yes | 249 | Yes | Yes^1^ | No | 395 | Yes | 400 | Yes | 5 | 3 |
| - Piqray, *alpelisib* | breast | NR | No | 582 | Yes | Yes^1^ | No | NR | No | 298 | No | 3 |  |
| - Retsevmo, *selpercatinib* | other | 294 | Yes | NR | No | NR | No | 665 | Yes | NR | No | 2 |  |
| - Rozlytrek, *entrectinib* | other | 300 | Yes | 399 | Yes | Yes^1^ | No | 564 | Yes | 358 | Yes | 5 | 0 |
| - Tukysa, *tucatinib* | breast | 468 | Yes | 538 | Yes | NR | No | NR | No | 507 | No | 3 |  |
| Reimbursable products |  | 6 | | 5 | | 3 | | 5 | | 5 | |  |  |
| Managed entry agreements |  | 6 | | 5 | | 0 | | 5 | | 2 | |  |  |
| Average days from authorization to reimbursement |  | 414 | | 477 | | - | | 562 | | 362 | |  |  |
| Average days from authorization to reimbursement in products with a managed entry agreement |  | 414 | | 477 | | - | | 562 | | 379 | |  |  |
| Authorized in 2019 | | | | | | | | | | | |  |  |
| - Erleada, *apalutamide* | prostatic | 744 | Yes | 1203 | Yes | Yes^1^ | Yes^2^ | 529 | Yes | 838 | Yes | 5 | 3 |
| - Lorviqua, *lorlatinib* | lung | 687 | Yes | 545 | Yes | Yes^1^ | No | 588 | Yes | 144 | Yes | 5 | 1 |
| - Talzenna, *talazoparib* | breast | NR | No | NR | No | Yes^1^ | No | 711 | Yes | 712 | Yes | 3 |  |
| - Vitrakvi, *larotrectinib* | other | NR | No | 409 | Yes | NR | No | NR | No | 409 | Yes | 2 |  |
| - Vizimpro, *dacomitinib* | lung | NR | No | NR | No | NR | No | 230 | Yes | 73 | Yes | 2 |  |
| - Xospata, *gilteritinib* | hematological | 922 | Yes | 359 | Yes | Yes^1^ | No | 922 | Yes | 699 | Yes | 5 | 2 |
| Reimbursable products |  | 3 | | 3 | | 4 | | 5 | | 6 | |  |  |
| Managed entry agreements |  | 3 | | 3 | | 1 | | 5 | | 6 | |  |  |
| Average days from authorization to reimbursement |  | 784 | | 438 | | - | | 596 | | 479 | |  |  |
| Average days from authorization to reimbursement in products with a managed entry agreement |  | 784 | | 438 | | - | | 596 | | 479 | |  |  |
| Authorized in 2018 | | | | | | | | | | | |  |  |
| - Alunbrig, *brigatinib* | lung | 370 | No | 376 | Yes | Yes^1^ | No | 396 | Yes | 84 | No | 5 | 1 |
| - Braftovi, *encorafenib* | other | 861 | Yes | 804 | Yes | Yes^1^ | No | 369 | Yes | 194 | No | 5 | 3 |
| - Mektovi, *binimetinib* | other | 1163 | Yes | 650 | Yes | NR | No | 368 | Yes | 193 | Yes | 4 |  |
| - Nerlynx, *neratinib* | breast | NR | No | 823 | Yes | NR | No | NR | No | 539 | No | 2 |  |
| - Rubraca, *rucaparib* | other | NR | No | NR | No | NR | No | NR | No | NR | No | 0 |  |
| - Verzenios, *abemaciclib* | breast | 147 | No | 97 | Yes | NR | No | 362 | Yes | 278 | No | 4 |  |
| Reimbursable products |  | 4 | | 5 | | 2 | | 4 | | 5 | |  |  |
| Managed entry agreements |  | 2 | | 5 | | 0 | | 4 | | 1 | |  |  |
| Average days from authorization to reimbursement |  | 635 | | 550 | | - | | 374 | | 258 | |  |  |
| Average days from authorization to reimbursement in products with a managed entry agreement |  | 1012 | | 550 | | - | | 374 | | 193 | |  |  |
| Authorized in 2017 | | | | | | | | | | | |  |  |
| - Alecensa, *alectinib* | lung | 734 | No | 653 | No | Yes^1^ | No | 494 | Yes | 282 | No | 5 | 2 |
| - Fotivda, *tivozanib* | other | 566 | No | 615 | Yes | NR | No | 613 | Yes | 93 | No | 4 |  |
| - Kisqali, *ribociclib* | breast | 244 | No | 587 | Yes | Yes^1^ | No | 164 | Yes | 163 | Yes | 5 | 1 |
| - Rydapt, *midostaurin* | hematological | 135 | Yes | 317 | Yes | Yes^1^ | No | 763 | Yes | 135 | No | 5 | 1 |
| - Zejula, *niraparib* | other | 1315 | Yes | 197 | Yes | Yes^1^ | No | 1383 | Yes | 745 | Yes | 5 | 2 |
| Reimbursable products |  | 5 | | 5 | | 4 | | 5 | | 5 | |  |  |
| Managed entry agreements |  | 2 | | 4 | | 0 | | 5 | | 2 | |  |  |
| Average days from authorization to reimbursement |  | 599 | | 474 | | - | | 683 | | 284 | |  |  |
| Average days from authorization to reimbursement in products with a managed entry agreement |  | 725 | | 429 | | - | | 683 | | 454 | |  |  |
| Authorized in 2016 | | | | | | | | | | | |  |  |
| - Ibrance, *palbociclib* | breast | Yes^1^ | No | 722 | Yes | Yes^1^ | No | 1076 | Yes | 234 | Yes | 5 | 2 |
| - Lonsurf, *trifluridine and tipiracil* | other | NR | No | 1285 | No | Yes^1^ | Yes^2^ | 483 | Yes | 152 | No | 4 |  |
| - Ninlaro, *ixazomib* | hematological | Yes^1^ | Yes | 802 | Yes | Yes^1^ | No | 1120 | Yes | 557 | Yes | 5 | 1 |
| - Tagrisso, *osimertinib* | lung | 1164 | Yes | 1004 | Yes | Yes^1^ | No | 1876 | Yes | 608 | Yes | 5 | 1 |
| - Venclyxto, *venetoclax* | hematological | 1436 | No | 1458 | Yes | NR | No | 995 | Yes | 513 | Yes | 4 |  |
| Reimbursable products |  | 4 | | 4 | | 4 | | 5 | | 5 | |  |  |
| Managed entry agreements |  | 2 | | 4 | | 1 | | 5 | | 4 | |  |  |
| Average days from authorization to reimbursement |  | 1300 | | 997 | | - | | 1110 | | 413 | |  |  |
| Average days from authorization to reimbursement in products with a managed entry agreement |  | 1164 | | 997 | | - | | 1110 | | 478 | |  |  |
| Authorized in 2015 | | | | | | | | | | | |  |  |
| - Cotellic, *cobimetinib* | other | NR | No | NR | No | Yes^1^ | No | Yes^1^ | N/A | NR | No | 2 |  |
| - Farydak, *panobinostat* | hematological | NR | No | NR | No | NR | No | 1249 | Yes | NR | No | 1 |  |
| - Lenvima, *lenvatinib* | other | 1779 | Yes | 949 | No | Yes^1^ | No | 1663 | Yes | 189 | No | 5 | 2 |
| - Odomzo, *sonidegib* | other | NR | No | 2452 | Yes | NR | No | 2208 | Yes | Yes^1^ | No | 3 |  |
| - Zykadia, *ceritinib* | lung | Yes^1^ | No | NR | No | Yes^1^ | No | 1209 | Yes | 223 | No | 4 |  |
| Reimbursable products |  | 2 | | 2 | | 2 | | 5 | | 3 | |  |  |
| Managed entry agreements |  | 1 | | 1 | | 0 | | 4^2^ | | 0 | |  |  |
| Average days from authorization to reimbursement |  | 1779 | | 1701 | | - | | 1582^2^ | | 201^2^ | |  |  |
| Average days from authorization to reimbursement in products with a managed entry agreement |  | 1779 | | 2452 | | - | | 1582^2^ | | - | |  |  |
| Authorized in 2014 | | | | | | | | | | | |  |  |
| - Cometriq, *cabozantinib* | other | NR | No | 1413 | Yes | NR | No | NR | No | 245 | No | 2 |  |
| - Imbruvica, *ibrutinib* | hematological | NR | No | 1137 | Yes | Yes^1^ | No | 419 | Yes | 235 | No | 4 |  |
| - Lynparza, *olaparib* | other | 1576 | Yes | 1143 | Yes | Yes^1^ | No | 307 | Yes | 71 | No | 5 | 2 |
| - Mekinist, *trametinib* | other | Yes^1^ | Yes | 2832 | Yes | Yes^1^ | No | Yes^1^ | N/A | 718 | No | 5 | 3 |
| - Vargatef, *nintedanib* | lung | NR | No | 314 | No | NR | No | NR | No | 182 | No | 2 |  |
| - Zydelig, *idelalisib* | hematological | NR | No | 1656 | No | NR | No | 424 | Yes | 160 | No | 3 |  |
| Reimbursable products |  | 2 | | 6 | | 3 | | 4 | | 6 | |  |  |
| Managed entry agreements |  | 2 | | 4 | | 0 | | 3^2^ | | 1 | |  |  |
| Average days from authorization to reimbursement |  | 1576 | | 1416 | | - | | 383^2^ | | 269 | |  |  |
| Average days from authorization to reimbursement in products with a managed entry agreement |  | 1576 | | 1631 | | - | | 383^2^ | | 718 | |  |  |
| Authorized in 2013 | | | | | | | | | | | |  |  |
| - Bosulif, *bosutinib* | hematological | Yes^1^ | No | 1101 | No | Yes^1^ | No | Yes^1^ | N/A | 191 | No | 5 | 1 |
| - Erivedge, *vismodegib* | other | NR | No | 324 | No | NR | No | Yes^1^ | N/A | Yes^1^ | No | 3 |  |
| - Giotrif, *afatinib* | lung | NR | No | 736 | No | NR | No | Yes^1^ | N/A | 212 | No | 3 |  |
| - Iclusig, *ponatinib* | hematological | Yes^1^ | No | 1857 | Yes | Yes^1^ | No | Yes^1^ | N/A | 635 | No | 5 | 3 |
| - Imnovid, *pomalidomide* | hematological | 2298 | Yes | 300 | Yes | Yes^1^ | No | Yes^1^ | N/A | 318 | Yes | 5 | 3 |
| - Stivarga, *regorafenib* | other | 1619 | Yes | 552 | No | Yes^1^ | No | Yes^1^ | N/A | 893 | No | 5 | 2 |
| - Tafinlar, *dabrafenib* | other | Yes^1^ | Yes | 340 | Yes | Yes^1^ | No | Yes^1^ | N/A | 186 | No | 5 | 1 |
| - Xtandi, *enzalutamide* | prostatic | 2896 | Yes | 437 | Yes | Yes^1^ | No | Yes^1^ | N/A | 740 | Yes | 5 | 2 |
| Reimbursable products |  | 6 | | 8 | | 6 | | 8 | | 8 | |  |  |
| Managed entry agreements |  | 4 | | 4 | | 0 | | - | | 2 | |  |  |
| Average days from authorization to reimbursement |  | 2271 | | 706 | | - | | - | | 454 | |  |  |
| Average days from authorization to reimbursement in products with a managed entry agreement |  | 2271 | | 734 | | - | | - | | 529 | |  |  |
| Authorized in 2012 | | | | | | | | | | | |  |  |
| - Caprelsa, *vandetanib* | other | NR | No | NR | No | Yes^1^ | No | Yes^1^ | N/A | 1289 | No | 3 |  |
| - Inlyta, *axitinib* | other | Yes^1^ | No | 516 | No | Yes^1^ | No | Yes^1^ | N/A | 151 | Yes | 5 | 2 |
| - Jakavi, *ruxolitinib* | hematological | NR | No | 1195 | No | Yes^1^ | No | Yes^1^ | N/A | 590 | No | 4 |  |
| - Zelboraf, *vemurafenib* | other | NR | No | 1018 | Yes | Yes^1^ | No | Yes^1^ | N/A | 1139 | No | 4 |  |
| - Xalkori, *crizotinib* | lung | Yes^1^ | No | 647 | Yes | Yes^1^ | No | Yes^1^ | N/A | 439 | No | 5 | 3 |
| Reimbursable products |  | 2 | | 4 | | 5 | | 5 | | 5 | |  |  |
| Managed entry agreements |  | 0 | | 2 | | 0 | | - | | 1 | |  |  |
| Average days from authorization to reimbursement |  | - | | 844 | | - | | - | | 722 | |  |  |
| Average days from authorization to reimbursement in products with a managed entry agreement |  | - | | 833 | | - | | - | | 151 | |  |  |
| Authorized in 2011 | | | | | | | | | | | |  |  |
| - Teysuno, *tegafur, gimeracil and oteracil* | other | NR | No | 414 | Yes | NR | No | Yes^1^ | N/A | 375 | No | 3 |  |
| Reimbursable products |  | 0 | | 1 | | 0 | | 1 | | 1 | |  |  |
| Managed entry agreements |  | 0 | | 1 | | 0 | | - | | 0 | |  |  |
| Average days from authorization to reimbursement |  | - | | 414 | | - | | - | | 375 | |  |  |
| Average days from authorization to reimbursement in products with a managed entry agreement |  | - | | 414 | | - | | - | | - | |  |  |
| Authorized in 2010 | | | | | | | | | | | |  |  |
| - Zytiga, *abiraterone* | prostatic | Yes^1^ | No | 209 | Yes | Yes^1^ | No | Yes^1^ | N/A | 1365 | Yes | 5 | 3 |
| - Votrient, *patsopanib* | other | NR | No | 201 | No | Yes^1^ | Yes^2^ | Yes^1^ | N/A | 180 | No | 4 |  |
| Reimbursable products |  | 1 | | 2 | | 2 | | 2 | | 2 | |  |  |
| Managed entry agreements |  | 0 | | 1 | | 1 | | - | | 1 | |  |  |
| Average days from authorization to reimbursement |  | - | | 205 | | - | | - | | 773 | |  |  |
| Average days from authorization to reimbursement in products with a managed entry agreement |  | - | | 209 | | - | | - | | 1365 | |  |  |
| All products in 2010–2021 | | | | | | | | | | | |  |  |
| Reimbursable products n, % of all 67 products |  | n=36, 54%^4^ | | n=47, 70% | | n=36, 54% | | n=50, 75% | | n=52, 78% | |  |  |
| Managed entry agreements n |  | 23 | | 35 | | 3^2^ | | 32 | | 19 | |  |  |
| Average days from authorization to reimbursement |  | 895^3^ | | 753 | | - | | 748^3^ | | 416^3^ | |  |  |
| Average days from authorization to reimbursement in products with a managed entry agreement (in comparison to previous) |  | 905^3^ (+10) | | 787 (+34) | | - | | 748^3^ | | 485^3^ (+69) | |  |  |

Diseases classified as haematological, lung, breast, prostatic and other (including medicines with multiple indications and/or indications other than haematological malignancies, or lung, breast or prostate cancer) according to the cancer type.

DtR = Days to reimbursement in some indication from the first marketing authorization date

MEA = Managed entry agreement

NR = not reimbursed

^1^ Dates not available

N/A = Information not provided by the respondent

^2^ Inpatient use only

^3^Calculated excluding the products with incomplete information

^4^ Before 2017, all products were made available and reimbursed if the company launched in Denmark. After the establishment of the Danish Medicines Council in 2017, all new hospital medicine must be approved by the Danish Medicines Council to become standard of care and to be reimbursed. The Danish Medicines Council make treatment guidelines for therapeutic areas and assessments of new medicines.

Hauté Autorité de Santé (HAS) evaluation: Clinical added value when compared with available therapies according to the French National Authority for Health [ref. 48]. Scale for clinical added value: great (5), significant (4), moderate (3), minor (2), no added benefit (1), not recommended for reimbursement (0). If not recommended for reimbursement, the treatment is not compared to available therapies. Clinical added value may vary according to the indication.
